# Compound Dislocation of the Os Naviculare of the Tarsus

**Published:** 1831

**Authors:** 


### Compound Dislocation of the Os Naviculare of the Tarsus.

Mr. Brodie in his lectures is accustomed to mention a case of dislocation of the os naviculare, which started upwards. The patient was afterwards subject to periodic pains in the course of the nerves. We are not aware that such a dislocation is mentioned by other surgeons, though no doubt it has occasionally occurred. The following case is not undeserving of notice.

A carman, ætat. 28, had his foot jammed between the curb-stone and a coach-wheel, occasioning a wound which bled freely. He was carried to a hospital in Paris. The wound extended from the tendo-achillis, below the ankle, to opposite the middle of the metatarsal bone of the great toe, whilst another wound commenced before and above the ankle, and passing forwards to join the former at an acute angle, formed along with it a flap. The wound was not deep, but a little anterior to its centre a bone projected presenting an articulating surface. There was a considerable depression on the outside of the foot, below the external malleolus; the lower part of the leg and the foot were much swollen. It was thought that there was dislocation of the astragalus, and some very forcible, indeed violent, efforts were made to reduce the bone thus supposed to be displaced, but in vain, and at length they were abandoned, and the foot enveloped in a poultice. Next morning the swelling was greater and had spread higher, and the skin in the neighbourhood of the wound threatened gangrene; the patient refused amputation. The appearances grew worse during the day, and in the evening a phlyctena existed on the posterior part of the leg. Amputation was now performed, but the patient died eight days afterwards with symptoms of pleuro-pneumonia, which dissection proved to exist. It was now discovered that the astragalus was perfectly in place, and that the depression below the external malleolus was merely a result of the swelling elsewhere. The os naviculare had been dislocated from the astragalus and cuneiform bones inwards; its external extremity, connected with the cuboid, had been fractured from behind forwards, and a small portion still remained attached by ligamentous fibres to the cuboid bone. We certainly cannot say much either for the diagnosis or the practice in this case.—*Hebdomadaire*, T. II. No. 19.

---

